# Anti-Tumor Activity of *Eurycoma longifolia* Root Extracts against K-562 Cell Line: *In Vitro* and *In Vivo* Study

**DOI:** 10.1371/journal.pone.0083818

**Published:** 2014-01-07

**Authors:** Omar Saeed Ali Al-Salahi, Dan Ji, Amin Malik Shah Abdul Majid, Chan Kit-Lam, Wan Zaidah Abdullah, Abdelhamid Zaki, Shah Kamal Khan Jamal Din, Narazah Mohd Yusoff, Aman Shah Abdul Majid

**Affiliations:** 1 Advanced Medical and Dental Institute (AMDI), Universiti Sains Malaysia (USM), Kepala Batas, Pulau Pinang, Malaysia; 2 Key Lab of Visual Damage and Regeneration & Restoration of Chongqing, Southwest Eye Hospital, Southwest Hospital, The Third Military Medical University, Chongqing, P.R. China; 3 School of Pharmaceutical Sciences, Universiti Sains Malaysia, Penang, Malaysia; 4 Haematology Department, School of Medical Sciences, USM, Kubang Kerian, Kelantan, Malaysia; 5 Therapeutic Chemistry Department, National Research Centre, Cairo University, Dokki, Cairo, Egypt; 6 OMF Surgery, Hospital Sultanah Bhiyah, Alor Setar, Kedah, Malaysia; Duke University Medical Center, United States of America

## Abstract

*Eurycoma longifolia* Jack has been widely used in traditional medicine for its antimalarial, aphrodisiac, anti-diabetic, antimicrobial and anti-pyretic activities. Its anticancer activity has also been recently reported on different solid tumors, however no anti-leukemic activity of this plant has been reported. Thus the present study assesses the *in vitro* and *in vivo* anti-proliferative and apoptotic potentials of *E. longifolia* on K-562 leukemic cell line. The K-562 cells (purchased from ATCC) were isolated from patients with chronic myelocytic leukemia (CML) were treated with the various fractions (TAF273, F3 and F4) of *E. longifolia* root methanolic extract at various concentrations and time intervals and the anti-proliferative activity assessed by MTS assay. Flow cytometry was used to assess the apoptosis and cell cycle arrest. Nude mice injected subcutaneously with 10^7^ K-562 cells were used to study the anti-leukemic activity of TAF273 *in vivo*. TAF273, F3 and F4 showed various degrees of growth inhibition with IC50 values of 19, 55 and 62 µg/ml, respectively. TAF273 induced apoptosis in a dose and time dependent manner. TAF273 arrested cell cycle at G1and S phases. Intraperitoneal administration of TAF273 (50 mg/kg) resulted in a significant growth inhibition of subcutaneous tumor in TAF273-treated mice compared with the control mice (*P* = 0.024). TAF273 shows potent anti-proliferative activity *in vitro* and *in vivo* models of CML and therefore, justifies further efforts to define more clearly the potential benefits of using TAF273 as a novel therapeutic strategy for CML management.

## Introduction

Chronic myelocytic leukemia (CML) is a malignant disease of the human hematopoietic stem cell which is characterized by marked increase in granulocytes bone marrow hyperplasia and spleenomegaly [1], [2]. CML accounts for 15–20 percent of all leukemias [1], [3] with a worldwide incidence of 1–2/100,000 [4], [5]. The Philadelphia chromosome which resulted in the bcr/abl gene rearrangement is the hallmark of this disease. It is present in more than 90% of CML cases [6]. Chemotherapy is always the first choice for CML patients. Imatinib, alone or in combination with other drugs, is successfully used in the treatment of CML. However, the emergence of resistant and the high relapse rate to imatinib bring difficulty to the treatment of CML [7], [8]. Therefore searching for new compounds becomes a necessity.

Natural products, either microorganisms or plants, are rich resources of anti-cancer agents. *E. longifolia* Jack, an evergreen flowering tree from a Simaroubaceae family, is a herbal medicinal plant of South-East Asia. In Malaysia it is known as ‘Tongkat Ali’ [9]. The plant is rich in various bioactive compounds such as eurycomaoside, eurycolactone, eurycomalactone, eurycomanone, 14,15 β-dihydroxyklaineanonen, eurycomanol and eurycomalactone, 13,21-dihydroeurycomanone, 13_α(21)- epoxyeurycomanone and an alkaloid, 9-methoxycanthin-6-one [10]. *E. longifolia* has shown anticancer activities on various solid tumors including lung, breast and cervical cancers [10], [11], [12] as well as anti-parasitic activity [13], [14]. To further explore its antileukemic activity *in vitro* and *in vivo*, human chronic myelocytic leukemia cell line (K-562) served as a model in this study.

## Materials and Methods

No specific permissions were required. The roots of E. longifolia. were identified and purchased in Perak, Malaysia by a pharmaceutical company, Hovid Berhad, in Ipoh. The field studies did not involve endangered or protected species. The study protocol was approved by Animal Ethics Committee, Universiti Sains Malaysia (USM), No: USM/Animal Ethics Approval/2010/(60) (254).

### Plant material

The roots of *E. longifolia* were identified and purchased in Perak, Malaysia by a pharmaceutical company, Hovid Berhad, in Ipoh. A voucher specimen (No. 785-117) was deposited in Penang Botanical Garden, Penang, Malaysia. The air-dried powdered roots of *E. longifolia* (11.6 kg) were extracted with 6×4 l of 95% MeOH for 6 days at 60°C. The combined MeOH extract was evaporated to dryness to yield 485 g of dark brown residue which was next chromatographed on a Diaion HP 20 column with a H_2_O–MeOH (1∶0–0∶1) gradient to yield 4 fractions (F 1–4).

### Cells and Medium

K-562 leukemia cells were purchased from ATCC. K562 cells were maintained in RPMI 1640 medium (Gibco Inc), supplemented with 10% fetal bovine serum, 100 U/L of penicillin and 80 U/L streptomycin (Sigma), at 37°C in a humidified atmosphere of 5% CO_2_. This medium used through all the experiment.

### Cell viability assay

Cell viability was assessed by MTS assay using MTS reagent (CellTiter 96® AQueous One Solution Cell Proliferation assay, Promega). Briefly, 2×10^4^ exponentially growing K562 cells were seeded in 96-well culture plates with various concentrations of TAF273, F3, F4, eurycomanone and imatinib in a volume of 100 µl. After 48 h incubation at 37°C, 20 µl of MTS were added to each well, and the samples were incubated for a further 3 h at 37°C. Plates were analyzed on a Tecan M200Pro multimode micro-plate reader at 492 nm. Based on the results of this assay, TAF 273 was selected for further investigations.

### Clonogenic Assay

Colony-forming assay is considered the most reliable dose-dependent index of cytotoxcity *in vitro* [15]. Survival fractions of K-562 cells treated with various concentrations of TAF 273, eurycomanone and imatinib were assessed using fibrin clot assay. Briefly, K-562 cells were suspended in RPMI medium containing fibrin (3 mg/ml) at cell density of 1000 cells/ml. 1 ml of this cell suspension was transferred into each well of 6-well culture plates followed by addition of 20 µl of thrombin (prepared at 50 NIH U/mL in 1% bovine serum albumin in 0.15 M NaCl). The plates were then incubated at 37°C in 5% CO_2_ for 1 h to allow the medium to solidify. Two fold serial dilutions of TAF273 (6.25, 12.5, 25, 50 and 100 µg/ml ), eurycomanone (1.5, 3, 6 and 12 µg/ml ) and imatinib (0.05, 0.1, 0.2 and 0.4 µg/ml ) were prepared in RPMI and 2 ml of each concentration of a given compound were added into a separate well. 2 ml of RPMI without any compound were added into the well indicated negative control. The plates then incubated for 48 and 72 h at 37°C in 5% CO_2_ for in a humid atmosphere. After the indicated intervals, RPMI was removed from each well and replaced with fresh RPMI without any compound. Change medium was carried out every 3 days for 2 weeks. At day 14, colonies of ≥50 cells were counted in each well using stereomicroscope. The plating efficiency (PE) and survival fraction (SF) were calculated as follows:
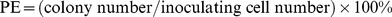


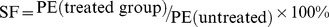
 [16].

A dose-survival curve was obtained for each experiment by plotting SF versus concentration.

### Annexin V-FITC/PI Assay

K-562 cells were exposed to TAF273 at various concentrations for various time points 24, 48, 72 and 96 h. Cells treated with staurosporine (1 µM) were acted as positive control while untreated cells were acted as a negative control. After each indicated time point, cells were harvested, washed and resuspended at (1×10^5^) with cold PBS. Apoptotic cells were identified using the Annexin V/PI Apoptosis Detection kit (BD PharmingenTM) according to the manufacturer's instructions. Flowcytometric analysis was performed within 1 h of staining. The amounts of early apoptosis and late apoptosis/necrosis were determined, respectively, as the percentage of Annexin V+/PI−or Annexin V+/PI+. Data acquisition and analysis were performed using a Becton Dickinson flow cytometer with CellQuest software.

### Hoechst 33342 staining

Morphological alterations indicating apoptosis were tested by Hoechst 33342 staining. Untreated and TAF273, eurycomanone and imatinib-treated K-562 cells (2×10^5^) were fixed with 4% paraformaldehyde for 20 min, washed twice with PBS and then incubated for 30 min with Hoechst 33342 (10 µg/ml) at 37°C in the dark. The cells were then observed using an inverted fluorescence microscope (AMG EVOS fI inverted microscope) and photographed. Nuclei with chromatin condensation or fragmented nuclei were counted and the apoptotic index was calculated.

### Cell Cycle Analysis

The Cycle TEST™ PLUS DNA reagent kit (BD biosciences, USA) was used for flow cytometry analysis of DNA content. Briefly, K-562 cells in exponential growth were treated with 25 and 50 µg/ml of TAF273 for 72 h. Treated and untreated cells (5×10^5^) were then washed three times with buffer solution. The cell pellet was incubated with 250 µl of trypsin-containing citrate buffer for 10 min at room temperature and then incubated with 200 µl of citrate buffer containing a trypsin inhibitor and RNase (10 min) before adding 200 µl of ice cold PI-containing buffer (125 µg/ml). Samples were analyzed on a Becton Dickinson FACScan flow cytometer and ModFIT software was used to determine the percentage of cells in the different phases of the cell cycle.

### RT^2^ profiler™ PCR array

To study the effect of treatment of K-562 cells with TAF273 on the apoptotic and cell cycle pathways, the human apoptosis RT^2^ profiler™ PCR array (Cat. No. PHAS-012) and human cell cycle RT^2^ profiler™ PCR array (Cat. No. PAHS-20) from SABiosciences were used. Each array consists of 84 pathway-related genes.

Total RNA was isolated from TAF273-treated and untreated K-562 cells using QIAamp RNA Blood Mini Kit (Qiagen, USA). Total RNA was measured on NanoVue spectrophotometer (GE Life Sciences, Sweden), the OD260/OD280 ratio was greater than 2.0 and the OD260/OD230 ratio was greater than 1.7. The RNA quality and integrity were also evaluated by experion bioanalyzer (Bio-rad, USA) using experion stdsens RNA analysis kit. RNA quality index (RQI) was 10. Experiments were performed according to the user's manual (Part # 1022A, Version 4.25, SABioscience Corporation). Briefly, 750 ng of total RNA were converted to cDNA using RT^2^ First Strand Kit (SABioscience, USA) and the PCR components mix was then prepared by adding, into a loading reservoir, 1350 µl of 2× RT^2^ SYBR Green Mastermix, 102 µl of cDNA synthesis reaction and 1248 µl of RNase-free water giving a total volume of 2700 µl. 25 µl of PCR components mix were dispensed into each well of the RT^2^ Profiler PCR Arrays and then placed in real time cycler (ABI 7300) for real-time detection. Expression levels of RNA were analyzed based on the Ct value.

### *In vivo* experiments

Eight-weeks-old male Balb/c nude mice from BioLASCO Taiwan were inoculated subcutaneously in the dorsal side with 10^7^ K562 cells, injected in 150 mL PBS solution. At day 8 of injection, animals were randomly assigned to control and treatment groups (n = 4). Mice in treatment group received TAF273 (50 mg/kg) via intraperitoneal injection while control group received only vehicle every other day for 16 days. Tumor dimensions were taken twice a week using digital caliper (TESA ShopCal, Swiss) tumor size and growth inhibition rate was calculated according to the following formulas:
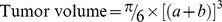
were *a* is longest diameter and *b* is the shortest [17]. 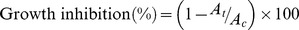 were *A_t_* is the mean tumor volume of treated mice and *A_c_* is the mean tumor volume of control mice [18].

The study protocol was approved by Animal Ethics Committee, Universiti Sains Malaysia (USM), No.: USM/Animal Ethics Approval/2010/(60) (254).

### Histology

To study the effect of TAF273 on the histological appearance, K-562 tumor xenograft samples were excised and fixed in 10% neutrally buffered formalin for 24 h and then embedded in paraffin. Paraffin blocks were prepared and sections of 5 µm were cut on a microtome (Accu-Cut® SRM™, SAKURA) and then were stained with hematoxylin and eosin.

### Statistical analysis

The differences between the means were analyzed by non-parametric Mann- Whitney test and student's *t* test using SPSS 11.5 software. P value less than to 0.05 was considered statistically significant.

## Results

### Assessment of viability

The cytotoxic potential of TAF273, F3 and F4 from *E. longifolia* root methanolic extract as well as eurycomanone (major constituent of TAF 273) and imatinib (positive control) was tested on K-562 cells. Treatment of K-562 cells for 48 h resulted in various degrees of growth inhibition as indicated by variation in the IC50 values (Table 1). The TAF273 showed a potent cytotoxic effect (IC50 = 19±3 µg/ml) while F3 and F4 showed a low cytotoxic activity (IC50 = 55±2 and 62±7 µg/ml, respectively). Eurycomanone also showed a strong antiproliferative activity with IC50 of 6±1 µg/ml. As can be seen from comparison of IC50 values, TAF273 and eurycomanone were less active than the imatinib, which showed a very strong activity against K-562 cells (IC50 = 0.2±0.05 µg/ml). TAF273 and eurycomanone cytotoxic activities were 95 and 30 times less, respectively.

**Table 1:** Half maximal inhibitory concentration (IC_50_) values of various fractions of *E. longifolia* root methanolic extract on K-562 cell line.

Test sample	IC_50_ (µg/ml)
TAF273	19±3
F3	55±2
F4	62±7
Eurycomanone	6 ±1
Imatinib	0.2±0.05

### Clonogenic assay

**Figure 1:**
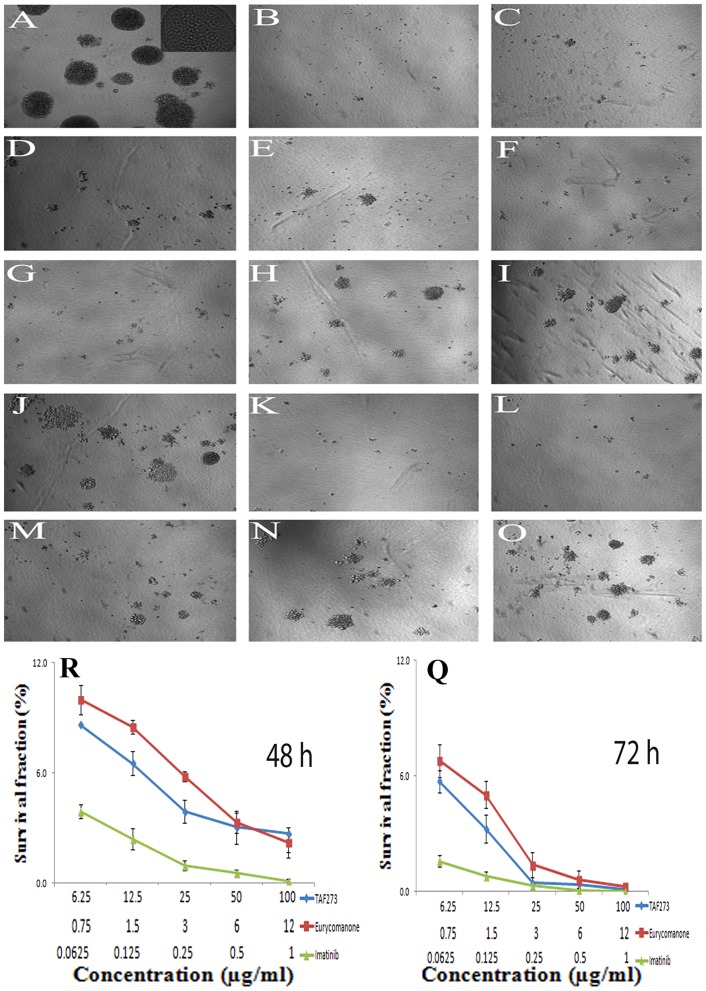
Colony morphology (upper panel) and Dose-response curves (lower panel). K-562 cells were treated for 48 h and 72 h with various concentrations of TAF 273, eurycomanone and imatinib (positive control). After the indicated time of treatment, cells were maintained without test materials up to 14 days. At day 14, the grown colonies were photographed and counted using light microscope. The percentages of survival fraction were then calculated. **A**): control (untreated cells); **B–E**): Imatinib (1, 0.5, 0.25 and 0.125 µg/ml, respectively); **F–J**): eurycomanone (12, 6, 3, 1.5 and 0.75 µg/ml, respectively); **K–O**): TAF273 (100, 50, 25, 12.5 and 6.25 µg/ml, respectively).

To confirm the cytotoxic effect of TAF273 and eurycomanone on K-562 cells, the colony formation assay was performed. The findings of this assay clearly showed that TAF273 and its major constituent, eurycomanone, have cytotoxic rather than cytostatic effect on K-562 cells. This is indicated by the decrease in the survival fractions (SF) even at concentrations lower than the IC50 values of TAF273 and eurycomanone. As shown in Figure 1R–1Q, the reduction in SF was dose and time dependent. The SF was less than 4% in the TAF273 and eurycomanone-treated cells for 48 h at concentrations of 25 and 6 µg/ml, respectively. The SF was further reduced to less 0.5% at the same concentrations when cells were treated for 72 h. At higher concentrations of both TAF273 and eurycomanone the SF was ≤0.1%. Figure 1A–1O showed that the size and the compactness of the colonies are also affected upon treatment.

### Assessment of apoptosis by Annexin-V assay

**Figure 2:**
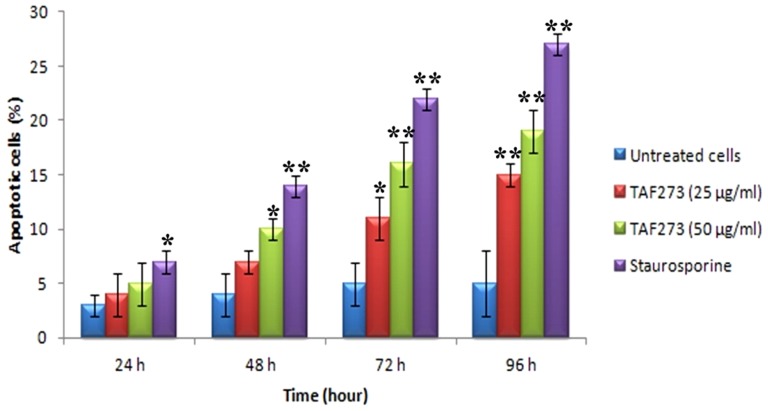
TAF273 induces externalization of phosphatidylserine. The histogram shows the apoptotic percentage of K-562 cells treated with TAF273 for various time points after staining with annexin V-FITC/PI stain. Staurosporine (1 µM)-treated cells were acted as positive control while untreated cells were acted as negative control. Values are expressed as mean ± SD. (*) indicates *P*<0.05 and (**) indicates *P*<0.01).

Annexin V/PI double parameter assay is a sensitive detection technique of apoptosis. The apoptotic cells were divided into two population; the early apoptosis (Annexin-V positive) and late apoptosis/necrosis (Annexin-V/PI positive). The treatment with TAF273 at concentrations of 25 and 50 µg/ml for various time points induced apoptosis in K-562 cells in a dose and time dependent manner. Statistically significant induction of apoptosis appeared at 48 h with 50 µg/ml TAF273 as compared with untreated cells (*P*<0.01). At 72 and 96 h, treatment with 25 µg/ml TAF273 increased the apoptotic population from 5±2% in the untreated cells to 11±1% and 15±1% (P<0.01 and <0.001), respectively. The treatment with 50 µg/ml increased apoptotic population to 16±2% and 19±2% (P<0.001), respectively. Staurosporine resulted in significant apoptosis as early as 24 h (*P*<0.01) as shown in Figure 2.

### Morphology of apoptosis by Hoechst staining

Nuclear changes such as chromatin condensation and DNA fragmentation are hallmarks of apoptotic cells. Effect of TAF273 and eurycomanone on nuclear morphology of K-562 cells was investigated by DNA-binding fluorescent dye (Hoechst 33342 stain).

**Figure 3:**
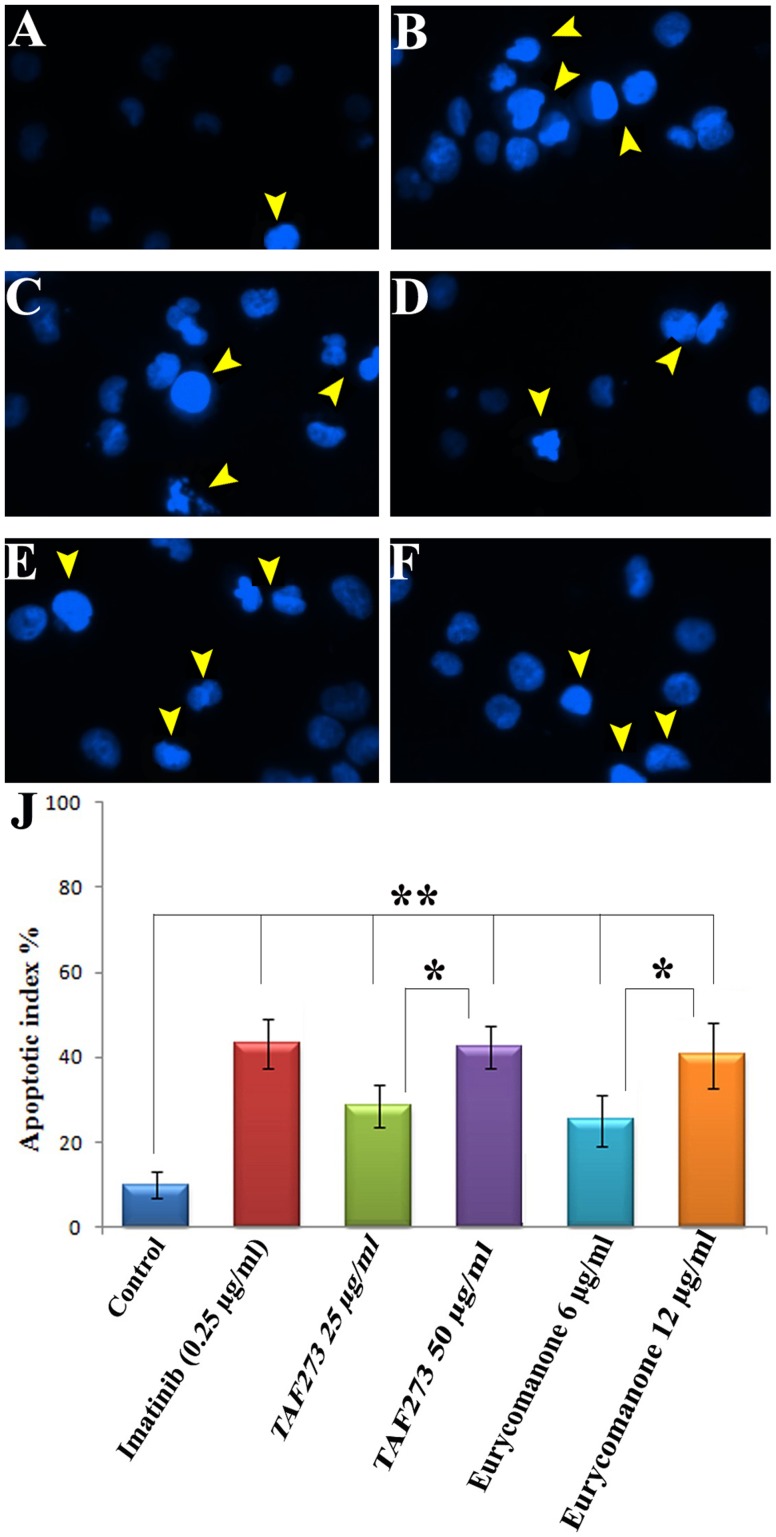
Nuclear morphology and apoptotic index of K-562 cells. K-562 cells were treated with TAF273 and eurycomanone and stained with Hoechst 33342 (10 µg/ml) for 96 h. K-562 cells treated with imatinib and untreated cells were acted as positive and negative control respectively. Cells with nuclear condensation are labeled with arrowheads Original magnification of 20×. Apoptotic index was calculated as: (the number of nuclei with chromatin condensation/the total number of nuclei)×100%. **A**: untreated cells; **B**: imatinib (0.25 µg/ml); **C**: TAF273 (50 µg/ml); **D**: TAF273 (25 µg/ml); **E**: eurycomanone (12 µg/ml); **F**: eurycomanone (6 µg/ml); **J**: apoptotic index. (*) indicates *P*<0.05 and (**) indicates *P*<0.01.

As shown in Figures 3B–3F, cells treated for 96 h with TAF273, eurycomanone and reference control (imatinib) showed remarkable changes in the chromatin structure including fragmentation, uniform condensation and forming clusters against the nuclear periphery with brighter fluorescence (arrowheads). In contrast, the untreated cells remained uniformly stained Figure 3A.

The apoptotic indexes were quantitated from the mean of at least three independent experiments. TAF273 at 25 µg/ml and 50 µg/ml increased the apoptotic index (AI) from approximately 10% in the untreated cells to approximately 30% and 41%, respectively. Furthermore, eurycomanone at 6 and 12 µg/ml increased the AI to 28% and 39%, respectively (Figure 3J). The difference in the AI between two concentrations of both TAF273 and eurycomanone was also statistically significant (*P*<0.05) indicating that the induction of apoptosis was dose dependent.

### Cell cycle effects

**Figure 4:**
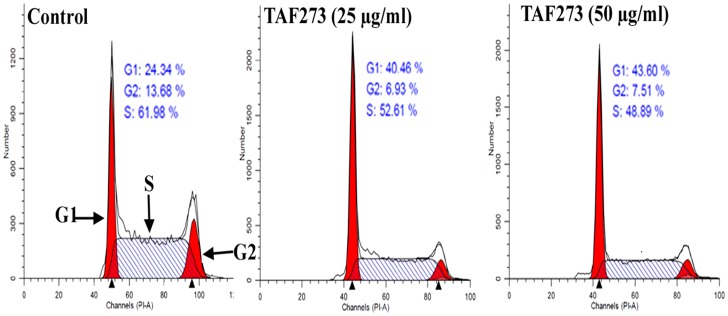
Effect of TAF273 on cell cycle distribution in K-562 cells treated for 72 h. The flow cytometry analysis of PI-labeled cells indicates that treatment of K-562 cells with 25 µg/ml of TAF273 for 72 h led to increase in the G1 population from 24.34% in the control cells to 40.48% in treated cells. In mean time, the S and G2 populations decreased from 61.98% and 13.68% in the control cells to 40.48% and 6.93% in treated cells, respectively. On the other hand, treatment with TAF273 (50 µg/ml) caused increase in G1 population from 24.34% to 43.60% in the control and treated cells, respectively. In contrast, S and G2 populations decreased from 61.98% and 13.68% in the control cells to 48.89% and 7.51% in the treated cells, respectively.

The flow cytometry analysis of PI-labeled cells indicates that treatment of K-562 cells with 25 µg/ml of TAF273 for 72 h led to increase in the G1 population from 24.34% in the control cells to 40.48% in treated cells. In mean time, the S and G2 populations decreased from 61.98% and 13.68% in the control cells to 40.48% and 6.93% in treated cells, respectively. On the other hand, treatment with TAF273 (50 µg/ml) caused increase in G1 population from 24.34% to 43.60% in the control and treated cells, respectively. In contrast, S and G2 populations decreased from 61.98% and 13.68% in the control cells to 48.89% and 7.51% in the treated cells, respectively (Figure 4).

### Gene expression determined by PCR array

**Figure 5:**
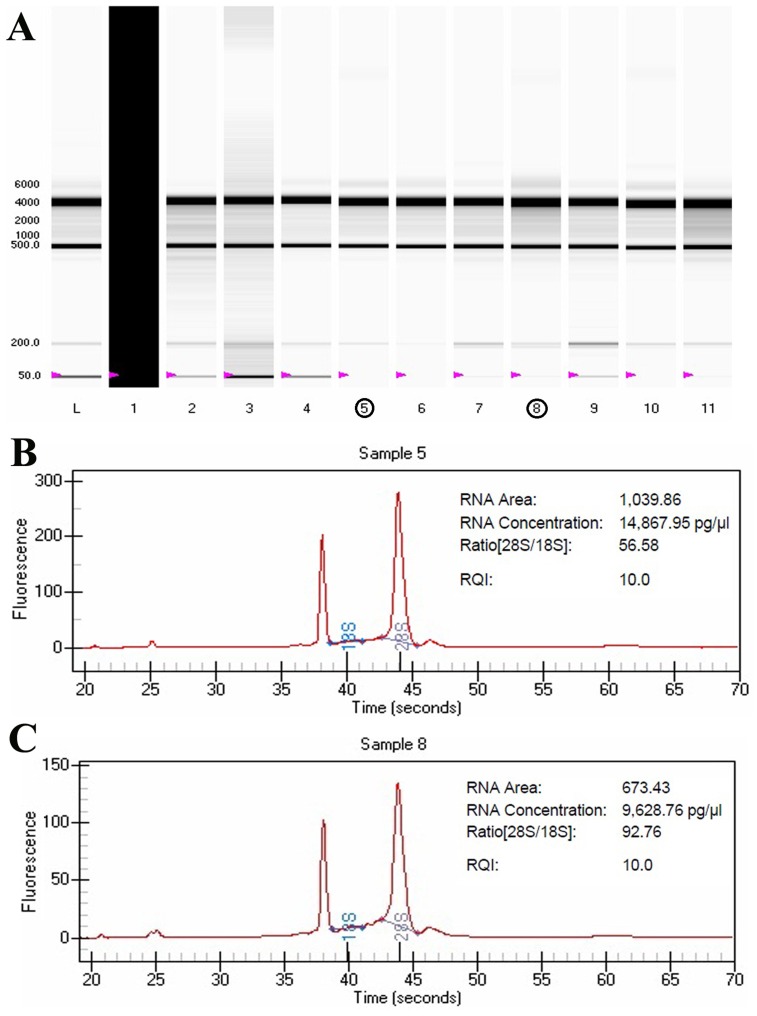
RNA quality and integrity. (**A**): virtual gel image showing Lane L: RNA ladder, Lane 1: no RNA sample, lanes 2–12 samples. Sample 5 and 8 were for the control and TAF273-treated cells, respectively. (**B**): electrophoregram for control sample; (**C**): electrophoregram for TAF273-treated sample.

**Figure 6:**
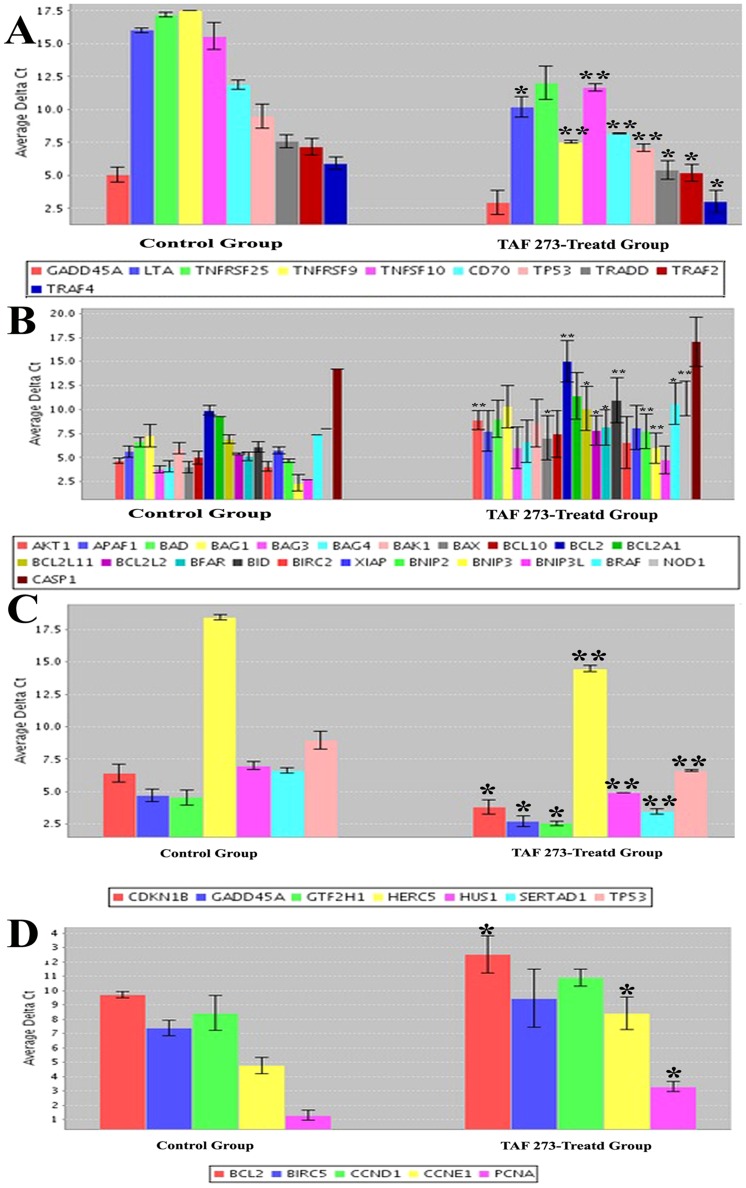
Expression changes of apoptosis and cell cycle related genes in K562 cells. Expression levels of RNA were analyzed based on the Ct value. (A): Up-regulated genes in apoptotic pathway; (B): Down-regulated genes in apoptotic pathway; (C): Up-regulated genes in cell cycle pathway; (D): Down-regulated genes in cell cycle pathway.

To improve the understating of the regulatory mechanisms leading to arrest of the cell cycle and apoptosis, the activation status of apoptosis and cell cycle pathways were analyzed using RT^2^ profiler™ PCR arrays. Since high quality of RNA samples is essential requirement for PCR array assays, RNA samples from control and TAF273-treated cells were analyzed by experion bioanalyzer and found to be of good quality as indicated by the RQI value of 10 (Figure 5). In Figure 6, expression levels of RNA were analyzed based on the Ct value. The higher Ct value indicates lower expression or down regulation of genes whereas the lower Ct value indicates high expression of genes. Analysis of apoptotic pathway showed that LTA, TNFRSF9, TNFSF10, CD70, TP53, TRADD, TRAF2 and TRAF4 genes were significantly upregulated (P<0.05 and P<0.01), and GADD45A and TNFRSF25 were non-significantly upregulated (P>0.05) (Figure 6A). Furthermore, AKT1, BCL2, BID, BNIP2, BNIP3 and NOD1 genes were significantly down-regulated (P<0.01), BAX, BCL2L11, BCL2L2, BFAR and BRAF were also significantly down-regulated (P<0.05) as shown in Figure 6B.

On the other hand, analysis of cell cycle pathway showed that CDKN1B, GADD45A and GTF2H1 genes were significantly up-regulated (P<0.05) and HERC5, HUS1, SERTAD1 and P53 (P<0.01) (Figure 6C). It was also found that, BCL2, CCNE1 and PCNA genes were significantly down-regulated (P<0.05) while BIRC5 and CCND1genes showed a non-significant down-regulation (Figure 6D).

### TAF273 inhibits tumor growth in a xenograft model of K-562 cells

**Figure 7:**
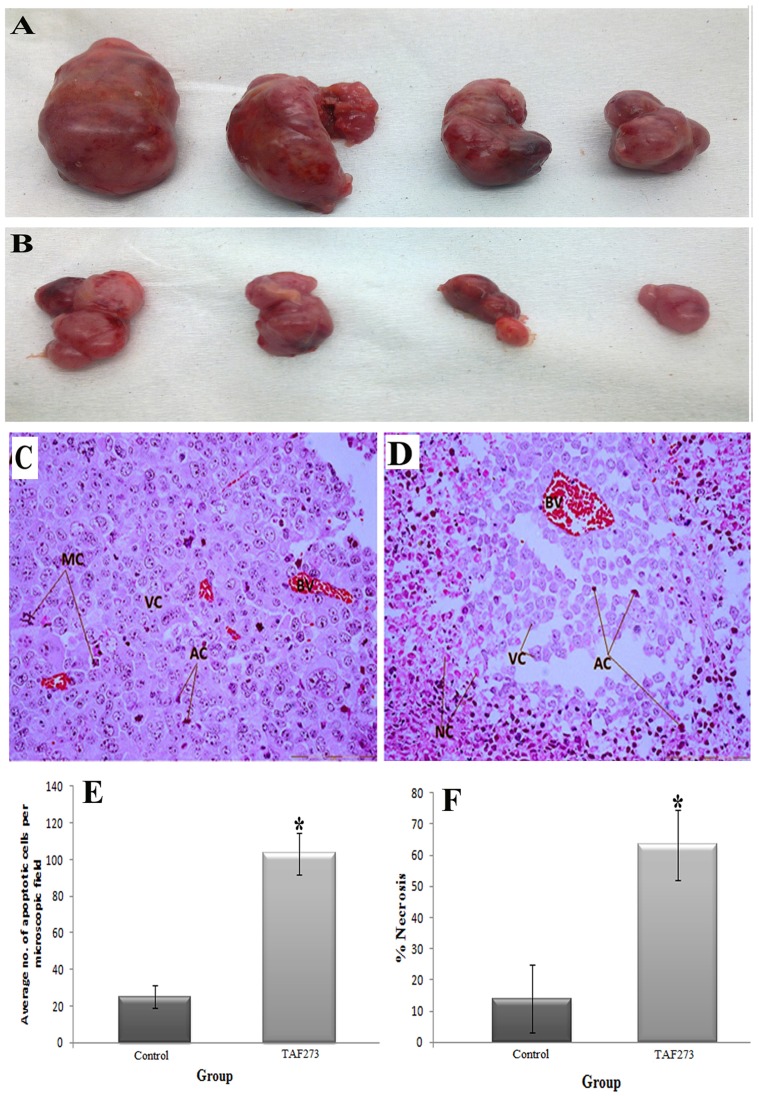
Effect of TAF273 on the size and histological appearance of subcutaneous tumor induced by injecting the K-562 cells in nude mice. (A) Gross appearance of tumors in the control mice. (B) Gross appearance of tumors in TAF273-treated mice. (**C**): An H&E-stained tumor section (original magnification of 40×) of the control group is composed of compact sheet of aggressively proliferating viable tumor cells (VC), abundance of blood vessels (BV), and the presence of mitotic figures (MC). (**D**): The tumor section (original magnification of 40×) of TAF273 (50 mg/kg) IP- treatment revealed notable changes in tumor histology, as significant loss of compact arrangement of viable tumor cells (VC), with less number of blood vessels (BV), abundance of apoptotic cells (AC) surrounded by necrotic regions (NC) and absence of mitotic figures. (**E**): Graphical comparison of the mean apoptotic cells/microscopic field (control vs TAF273). (**F**): Graphical comparison of the mean necrotic areas (control vs TAF273) as calculated by using imageJ softwere. Values are presented as mean ± SD, (*n* = 4).

Balb/c nude mice were injected subcutaneously with K-562 cells into the dorsal right side and allowed to grow for 8 days. The effect of the intraperitoneal administration of TAF273 (50 mg/kg) and the vehicle on the tumor growth was monitored by measuring tumor size twice a week. After 16 days of treatment, TAF273 significantly inhibited tumor growth by 85% compared with control (P<0.02) (Figures 7A–7B). This result clearly indicated that TAF273 has significant antiproliferative activity on K-562 cells growth *in vivo*.

The hematoxylin/eosin staining of tumor samples from control mice showed a compact and rounded shape of the viable tumor cells (VC), which were characterized by a very dense cell distribution with some mitotic figures (MC). Some apoptotic cells (AC) were also seen and characterized by the dark eosinophilic cytoplasm and dense purple nuclear chromatin (Figure 7C). Unlike control samples, tumor samples from TAF273-treated mice showed a significant reduction in the number of the viable tumor cells, increased number of apoptotic cells and increase in necrotic cells (NC) which were characterized by eosinophilic cytoplasm and no nuclei. Furthermore, the blood vessels were noticeably reduced and the mitotic figures were also not detected in TAF273-treated tumor samples (Figure 7D). The ImageJ software was used to calculate the percentage of necrosis in control versus TAF273 groups and was significantly higher in TAF273 group (*P*<0.05) (Figure 7F). The mean count of the apoptotic cells were 25±6 and 103±11 in the control and TAF273 groups, respectively (*P*<0.05) (Figure 7E).

## Discussion

Various strategies have been deployed to develop chemopreventive drugs over the last decade [19], [20]. More recently agents such as botanical drugs with the potential to modulate multistage carcinogenesis have been the focus. Although the science and regulatory requirements behind the development of botanical drugs as therapeutics of the future still remains a great challenge, the number of preliminary investigations into potential botanicals with chemopreventive properties, have been increasing exponentially [21]. Here we investigate the various isolates and purified Eurycomane, an active compound from the roots of *Eurycoma longifolia* and examine their cytotoxic effect in K-562 cells (purchased from ATCC) isolated from patients with chronic myelocytic leukaemia (CML). Their mechanism of action in inducing apoptosis *in vitro* and their ability to inhibit tumorogenesis in a mouse tumor model was also investigated. TAF273 in particular showed potent anti-proliferative activity in both *in vitro* and *in vivo* models.

The induction of apoptosis has been recognized as a strategy for the identification of anticancer drugs [22]. There is substantial evidence that alteration in the cellular and molecular pathways that control the cell cycle and apoptosis may change the sensitivity and resistance to anticancer agents [23]. There is an increasing realization that chemotherapeutic agents act primarily by inducing cancer cell death through the mechanism of apoptosis [24].

Previous studies have shown that eurycomanone exerted anti-proliferative activity in HepG2 cells, Hela cells, MCF-7 cells and lung cancer cells [23], [24], [25]. This supports the established mechanism how chemotherapeutic agents induce cancer cell death. It was shown that eurycomanone was more selective than tamoxifen which may potentially reduce the side effects commonly associated with chemotherapy. Other studies also demonstrated the positive effects of eurycomanone on noncancerous MDBK and Vero cells [26] and also noncancerous breast cells (MCF-10A) [12].

In the present study, we provide evidence to support eurycomanone as an apoptosis inducer in the human CML cancer cell line. Hoechst staining of DNA revealed that treatment with TAF273 and eurycomanone induced DNA fragmentation in K-562 cells. Inter-nucleosomal DNA fragmentation is the primary biochemical characteristic to indicate an early event of apoptosis and it represents a point of no return from the path to cell death. Furthermore there was clear morphological evidence to show shrinkage of cells, DNA condensations, nuclear and plasma membrane convulsion and nuclear fragmentation in K-562 cells treated with TAF273 and eurycomanone. These morphological changes were noted in K-562 cells exposed to both *E. longifolia* extract and eurycomanone. When stained with a Hoechst nuclear fluorochrome, the chromatin of the eurycomanone-treated K-562 cells can be seen to condense into lumps, thus exhibiting the punctuated morphology typical of apoptotic cells. In addition there was an increase in the number of apoptotic cells in TAF273 and eurycomanone-treated group. The magnitude of change was relatively significant in comparison to the growth inhibition. The distribution of cells in the population was seen to increase while the percent of apoptotic cells rising significantly. It is also evident that the G1 and S phase became the dominant phase in cells treated with TAF273 and eurycomanone. This occurred in a time-dependent manner with a late apoptosis predominating. These findings indicate that at the ranges of concentration studied, the anti-proliferative effect of TAF-273 and eurycomanone on K-562 cells could be attributed primarily to the induction of G1 and S arrest i.e. mostly targeted at cell division and DNA synthesis. Interestingly in a previous study, it was shown that eurycomanone, a major constituent of TAF273, arrested HpG2 cells at G2/M phase after 72 h [23]. Treatment for 48 and 72 h resulted in the block of treated cells at G1 and S phases. It was also previously reported that quassinoids prevent purine synthesis and inhibit DNA/RNA synthesis which may contribute to the prolonged G1/S arrest [27].

The cytotoxic effect is most likely attributed to the quassinoids compound particularly eurycomanone. Eurycomanone was found to account for approximately 20% (w/w) of TAF273 constituents [28]. Other quassinoid compounds isolated from TAF273 include 13,21-dihydroeurycomanone (0.72±0.06%), 13α(21)-epoxyeurycomanone (7.39±0.17%) and eurycomanol (9.54±0.22% w/w) [29]. Other compounds isolated include 2,3-dehydro-4α-hydroxylongilactone, 2,3-dihydroxy-1-(40-hydroxy-30-methoxyphenyl)-propan-1-one and scopolin [30]. Eurycomanone showed remarkable growth inhibitory effects against K-562 and HL-60 cells with IC50 values of 6±1 and 3.5±1, respectively. These results confirmed the assumption that, the cytotoxic effect of TAF273 comes mostly from eurycomanone.

Recent *in vitro* studies support the role of cytochrome C which participates in the activation of programme of cell death. Although normally residing in the mitochondrial inter-membrane space, exogenous cytochrome C when added to cytosol can still induce various apoptotic signals in a cell-free apoptosis system [31], i.e. studies have shown that microinjection of cytochrome C to cytosol also results in the induction of apoptosis [32]. Bcl-2 is another integral membrane protein located mainly on the outer membrane of mitochondria. As a pro-surivival protein its over-expression functions to prevent the induction of apoptosis in response to various biological stimuli. Since cytosolic cytochrome C release is vital for the initiation of the apoptosis, various *in vitro* studies have implied the possible connection between Bcl-2 and cytochrome C. Cells undergoing apoptosis were found to have an elevation of cytochrome C in the cytosol and a corresponding decrease in the mitochondria. Over-expression of Bcl-2 prevented the efflux of cytochrome C from the mitochondria and the initiation of apoptosis [33], [34].

Upon activation in response to cellular stress or DNA damage, the p53 tumor suppressor induces the expression of gene products involved in cell cycle arrest and apoptosis where Bax being an important downstream mediator of p53 [35]. It is thus reasonable to hypothesize that cytochrome C maybe involved in P53-induced apoptosis. In addition, Bcl-2 may regulate apoptosis by controlling cytochrome C release. Hence we can postulate that the inhibitory effect of eurycomanone on Bcl-2 ultimately may lead to an increase in the content of cytochrome C, which once released, directly activates the caspase dependent apoptosis and enhances apoptosis. It is also probable that eurycomanone may have a direct influence on Bax protein since increased levels of Bax protein have been shown to be able to directly induce release of cytochrome C conceivably by forming a pore in the outer membrane of mitochondria that allow cytochrome C to leak out [23].

Our study focused on TAF-273 induced apoptosis in K-562 cells and in addition our data also supports the previous reported work elucidating the mechanism of action of apoptosis induction in other cell lines. Furthermore we demonstrated via analysis of apoptotic pathway that various other genes regulating apoptosis and cell survival i.e. LTA, TNFRSF9, TNFSF10, CD70, TP53, TRADD, TRAF2 and TRAF4 were also significantly up-regulated. Profiling studies showed AKT1, BCL2, BID, BNIP2, BNIP3, NOD1, BAX, BCL2L11, BCL2L2, BFAR and BRAF were significantly down-regulated. With regard to cell cycle regulation, CDKN1B, GADD45A and GTF2H1, HERCS, HUS1, SERTAD1 and P53 genes were significantly up-regulated. It was also found that BCL2, CCNE1 and PCNA genes were significantly down-regulated.

Down regulation of cyclin-dependent kinase inhibitor 1B (CDKN1B or also known as p27) is reported in approximately 60% of human cancer and also found to be associated with poor prognosis and resistance to chemotherapeutic drugs. It also functions as a tumor suppressor [36]. It was found that, normal and tumor cells from p27-deficient mice showed impaired G2/M arrest after low doses of ionizing radiation [36]. GADD45A (Growth arrest and DNA damage-45 alpha) is a member of GADD45 family genes that are induced by genotoxic stress [37], [38]. GADD45 proteins are involved in cell cycle arrest, cell survival, DNA repair and apoptosis. GADD45A inhibits cdc2/cyclinB1via p38 pathway [37], [39]. Its role in S and G2/M arrest was previously reported [40], [41].

The p53 (tumor suppressor) as previously mentioned, is found to mediate cell cycle arrest and apoptosis in response to DNA damage, thus gained the name “guardian of the genome” [42]. Previous studies revealed that p53 mediate cell arrest at late G1phase [43], G0/G1 phase [44], and also G2/M phase [42]. *Hus1* gene was found to be crucial for an S-phase cell cycle checkpoint which inhibits DNA synthesis in response to genotoxins [45]. Together with the checkpoint rad proteins, Hus 1 protein mediate S phase arrest, upon exposure to genotoxic stress, via activation of cds1 kinases [46].

In this study, the investigation of gene expression of cell cycle pathway showed that treatment of K-562 cells with TAF273 resulted in increased expression of all of these genes mentioned above which may explain the block of the cells at the G1, G2 and S phase. The treatment with TAF273 also showed down-regulation of BCL2, CCNE1, PCNA, BIRC5 and CCND1 genes. The BCL2 molecule encoded by BCL2 gene has a dual function [47], it act as anti-apoptotic as well as anti-proliferative molecule.

It has been reported that BCL2 enhances G0 arrest and therefore contributing to cell cycle delay [47]. In this study, the BCL2 gene was down regulated which may suggest its role in induction of apoptosis rather in cell arrest. Cyclin E1 (CCNE1) is a positive regulator of the cell cycle. It is essential for the G1-S phase transition in normal dividing cells [48]. Over expression of CCNE1 in many human tumors including leukaemia, breast cancer and others, has been reported [49]. Treatment with TAF273 induced low expression of CCNE1 in K-562 cells which may be another explanation for the G1 phase arrest.

Proliferating cell nuclear antigen (PCNA) acts as an essential component of the replication and repair machinery in nucleic acid metabolism [50]. The binding of PCNA to cell cycle regulators such as CDK inhibitor, p21 (waf1/cip1) blocks its activity required for DNA replication [51]. Sequestering of PCNA by p21 is required for p53-dependent G1 arrest in damaged cells [52]. Based on these information it could be suggested that G1 phase arrest occurred in TAF273-treated K-562 cells may be attributed to the reduced PCNA gene expression. G1/S-specific cyclin-D1 (CCND1 or Cyclin D1) plays an essential role in the transition of cell from G1 to S phase through its reaction with CDK4 and CDK6 [53]. The down regulation of this gene may also contribute in the G1 phase arrest.

Animal models of cancer have been widely used to investigate the pharmacological effects of synthetic or naturally occurring agents. To confirm the anti-tumor effect of TAF273 *in vivo*, K-562 cells were inoculated into the mice to create subcutaneous tumors. Subsequently, the tumor-bearing mice were treated with TAF273 via oral and IP routes. This is the first study in which animal model (nude mice) was used to assess the anti-tumor activity of extract from *E. longifolia* in particular TAF273. The results showed that, the oral treatment was less effective than the IP injection (data not shown). In the former, the dose was 4 times higher than that used in IP injection; however, the growth inhibition rate was not statistically significant (*P*>0.05, data not shown). This may be due to the reduced bioavailability of effective compound in the plasma. In previous study, it was shown that the plasma concentration of the quassinoid after oral administration was much lower than after intravenous application. The study also indicated that eurycomanone was poorly bio-available when given orally [54]. Since the eurycomanone is stable at pH 1 [55] and poorly available in plasma after oral administration, it is then most likely subjected to first pass effect. In contrast to oral administration, IP injection of TAF273 significantly inhibited the tumor growth with growth inhibition rate of 80.8% (*P* = 0.021). This result was comparable with that of imatinib which showed an inhibition rate of 85.1%.

On the light of these findings, it can be concluded that taking TAF273 via IP or intravenous route is recommended to avoid the pre-systemic metabolism of the active compound and therefore to attain better anti-tumor effect. Histological examination of tumor sections from TAF273 mice also confirmed the *in vitro* findings regarding the induction of apoptosis. The apoptotic rate in the TAF273-treated group was approximately 4 times higher than that in the control group (*P*<0.001). Furthermore, the number of blood vessels was also significantly reduced in the TAF723-treated group which in turn could be the reason behind the increase in the necrosis rate in the test group. Hence based on the results of the *in vitro* and *in vivo* anti-tumor activity studies, it could be concluded that TAF273 has a promising anti-leukaemic activity.

In summary, TAF273 induced a group of cellular responses, including, inhibition of proliferation, and induction of apoptosis, cell cycle arrest and inhibition of blood vessels formation. It is likely that TAF-273 and eurycomanone inhibit cells growth at G1 and S phase and prevent cells from undergo mitosis process via apoptosis and TAF-273 enhances apoptosis in K-562 cells in a p53-dependent pathway. The large number of p53 expression induces translocation of Bax from cytoplasm into mitochondrial which lead to cytochrome C release and induces the apoptosis process to occur. Despite the current finding of p53 involvement in TAF-273 cytotoxicity, the exact downstream target of p53 is unclear and possibly involving multiple pathways, which is worthy of further investigation. Nevertheless all these events interfere with the tumor progression. These findings increase the present level of understanding of the mechanisms of action of TAF273, explaining its potent antitumor effect *in vitro* and *in vivo* models of CML. This study certainly justifies further efforts to define more clearly the potential benefits of using TAF273 in novel therapeutic strategies for CML.
